# Associations Among Binge Eating Behaviors, Emotion Regulation, and Chronotype in Female Greek University Students

**DOI:** 10.2174/011570159X431924251127110942

**Published:** 2026-01-13

**Authors:** Efi Karakosta, Evangelia-Maria Tsapakis, Anastasia Konsta, Anat Lan, Haim Einat, Konstantinos N. Fountoulakis

**Affiliations:** 1 3^rd^ Department of Psychiatry, School of Medicine, Faculty of Health Sciences, Aristotle University of Thessaloniki, Thessaloniki, Greece;; 2 1^st^ Department of Psychiatry, Papageorgiou General Hospital of Thessaloniki, Aristotle University of Thessaloniki, Thessaloniki, Greece;; 3 School of Behavioral Sciences, Tel Aviv-Yaffo Academic College, Tel Aviv, Israel

**Keywords:** Binge eating behaviors, eating disorders, emotion regulation, chronotypes, evening chronotype, circadian rhythm

## Abstract

**Introduction:**

This study aimed to examine the associations among binge eating behaviors (BEBs), emotion regulation (ER), and chronotypes, and whether ER mediates the relationship between chronotypes and BEBs in Greek female university students, a population in which these associations have not previously been investigated.

**Methods:**

This study was approved by the Research and Ethics Committee of the Medical School of Aristotle University of Thessaloniki (No. 91/23, dated 17/02/2023). A total of 269 female students (mean age (±SD) = 21.07 (±4.36)) from Aristotle University of Thessaloniki participated in this cross-sectional study. Participants completed a sociodemographic questionnaire and four validated instruments: the Eating Disorder Examination Questionnaire, the Binge Eating Scale, the Difficulties in Emotion Regulation Scale, and the Morningness-Eveningness Questionnaire.

**Results:**

BEBs were significantly correlated with eating psychopathology (rs = 0.723, *p <* 0.001) and with difficulties in emotion regulation (ER) (rs = 0.429, *p <* 0.001). ER difficulties were also associated with eating psychopathology (rs = 0.434, *p <* 0.001). An evening chronotype was significantly associated with greater ER difficulties (rs = –0.150, 0.014). Mediation analysis indicated that ER difficulties fully mediated the relationship between eveningness and BEBs (Indirect Effect = –0.06, 95% CI: –0.11 to –0.01).

**Discussion:**

ER difficulties are associated with both BEBs and eveningness. While eveningness is not directly associated with BEBs, it is indirectly related through ER difficulties, suggesting a potential psychological mechanism underlying this relationship.

**Conclusion:**

ER difficulties may be a key mechanism linking evening chronotype to BEBs. Future studies could explore whether interventions targeting ER and circadian alignment are beneficial for managing BEBs among individuals with an evening chronotype.

## 1 INTRODUCTION

 Binge eating disorder (BED) is the most prevalent eating disorder, characterized primarily by recurrent binge eating episodes [1-3]. BED is the most common eating disorder across both sexes [2, 4], although it is more prevalent among women [1, 5]. The one-year prevalence of BED among women is 1.6% and among men 0.8%, while the lifetime prevalence of BED among women is 3.6% and among men 2.1% [1]. BED frequently co-occurs with various psychiatric disorders, such as bipolar disorder, depression, anxiety disorders, and substance use disorders [1], as well as several somatic medical conditions and diseases [6, 7]. Consequently, BED patients experience a lower quality of life compared to the healthy population [8].

BED frequently co-occurs with obesity, with studies indicating that individuals with BED are more likely to be obese than those without eating disorders [9-11]. People with BED typically have higher body mass index (BMI) values, resulting in numerous adverse side effects, including increased risk for cardiometabolic diseases, type 2 diabetes, and dyslipidemia [12]. BMI offers several strengths as a screening tool for obesity in BED patients, including its ease of calculation, widespread clinical availability, and established associations with health risks in population studies. However, BMI cannot distinguish between fat and lean mass, fails to account for fat distribution patterns, and shows variable accuracy across different ethnic groups [13]. Therefore, a more comprehensive approach that combines anthropometric measures with clinical and psychological assessments is essential for effective diagnosis and treatment of BED.

The etiology of BED is complex and influenced by a combination of biological, neurochemical, environmental, social, and psychological factors [7, 14]. Furthermore, a genetic component is suggested, as it tends to occur within families [1].

One factor that contributes to both the development and maintenance of BED is emotion regulation [15]. ER encompasses a range of processes for monitoring, evaluating, and modifying the emotional state [16], involving strategies, adaptive or maladaptive, to regulate the intensity, duration, and expression of emotions [17, 18].

Maladaptive handling of emotions, particularly negative ones, signifies difficulties in ER, which are often manifested in eating psychopathology and are observed in BED patients [19, 20]. Existing literature on BED suggests a correlation between dysregulation in ER mechanisms and BED symptoms [21-24]. BED patients often report experiencing discomfort and negative emotions and express a desire to alleviate these feelings promptly [25]. However, not having easy access to adaptive ER strategies seems to lead individuals with BED to use food for managing and soothing these negative emotions [20]. Therefore, it is not only the experience of negative emotions, but also the lack of adaptive ER strategies that contribute to binge eating behaviors [26].

Existing literature suggests a possible relationship between circadian rhythms and BED. For instance, disturbances in circadian patterns, including irregular sleep-wake cycles and altered metabolic processes, may disrupt natural appetite regulation and contribute to disordered eating patterns seen in BED [27]. These disturbances are thought to affect the timing and intensity of hunger and satiety signals, leading individuals to experience a tendency toward nighttime eating and binge episodes [28].

One way to explore the interactions between circadian rhythms and BED is by examining chronotypes. Chronotypes refer to individuals' preferences for activity and sleep patterns across the day-night cycle. These preferences are determined by internal circadian processes and are modified by environmental factors, such as exposure to natural light, social schedules, and lifestyle choices [29]. Chronotypes reflect individual differences in preference for circadian rhythms, indicating variations in individuals' daily hormonal, physiological, and behavioral organization. Chronotypes can be evaluated using various methods and tools and are, in general, continuous variables, but it is common to define three main types in the general population: morning types, intermediate types, and evening types [30, 31]. Moreover, chronotype follows a developmental trajectory, with morningness prevailing in childhood, a shift toward eveningness in adolescence, and a gradual return to morningness by age 50 [32].

Numerous studies suggest that evening-type individuals, who prefer to be active later in the day and at night, are generally at a disadvantage compared to morning- and intermediate-types [33-36]. This disadvantage is observed across several parameters, including a greater propensity for psychiatric disorders, especially mood disorders [37], as well as reduced well-being among psychiatric patients [38].

In this context, the associations between chronotypes and BED were also examined, and it has been suggested that people with the evening chronotype are predisposed to obesity and BED [39]. Harb *et al*. (2012) [40] demonstrated a correlation between BED and chronotypes, with binge eating episodes more likely to occur in people leaning towards the evening chronotype. Moreover, Romo-Nava *et al*. (2020) [41] examined the link between the evening chronotype and eating disorders in patients with bipolar disorder and found that the evening chronotype group had poorer dietary habits and higher rates of binge eating, bulimia, and nighttime eating, highlighting the role of the circadian system in disordered eating. Furthermore, in a recent study, Lan *et al*. (2025) [42] reported that body image concerns can moderate the impact of chronotype on eating disorder risk, underscoring the importance of psychological factors in circadian–eating associations. Interestingly, chronotypes are also linked with ER. Specifically, ER was shown to be less adaptive in evening chronotypes [43], and individuals with an evening chronotype tend to use maladaptive ER strategies more frequently than adaptive strategies, which are more commonly observed in morning chronotypes [37]. Recent findings have further suggested that the association between chronotype and weight-related outcomes may operate through emotion regulation strategies and emotional eating. Specifically, Keenan *et al*. (2025) [44] reported that eveningness predicted higher BMI indirectly *via* greater use of maladaptive ER strategies and emotional eating, emphasizing the role of regulatory processes in circadian–eating links.

Connections between binge eating behaviors, chronotypes, and ER have been demonstrated in prior studies. However, this relationship has not yet been examined in Greece. Thus, this study was designed to delineate the association between binge eating behaviors, difficulties in ER, and chronotypes in Greece, specifically among Greek female university students, since eating psychopathology is more prevalent among women [1, 5]. Our hypotheses were: (1) binge eating behaviors would be associated with ER difficulties; (2) binge eating behaviors would be associated with chronotype, with eveningness correlated with higher binge eating scores; (3) eveningness would be associated with greater ER difficulties; and (4) ER difficulties would mediate the relationship between chronotype and binge eating behaviors.

## 2 MATERIALS AND METHODS

### 2.1 Participants

 The Research and Ethics Committee of the Medical School of Aristotle University of Thessaloniki approved the study (No. 91/23, dated 17/02/2023), and all procedures were conducted in accordance with the ethical standards of the 1964 Helsinki Declaration and its later amendments. All participants received study information and signed an informed consent form. Our sample consisted of 269 women studying at the Aristotle University of Thessaloniki (AUTh), the majority of whom were psychology students. Although the exact number of psychology students was not recorded, they represented the dominant field of study in our sample. Their participation in this research was voluntary. Inclusion criteria included being a woman, being a student at AUTh, not having visual or writing impairments, and being able to read and understand Greek. Conversely, exclusion criteria were male sex, not being enrolled as a student at AUTh, inability to comprehend Greek, or the presence of visual or writing impairments that could interfere with the reliable completion of questionnaires. These criteria were applied to ensure accurate self-reporting and data validity. Female students were recruited explicitly because eating disorders and binge eating behaviors are significantly more prevalent among women [1, 5], which allowed us to focus on a higher-risk group and achieve sufficient statistical power for detecting associations. Restricting the sample to AUTh students ensured a homogenous study population, facilitated recruitment in a controlled academic setting, and minimized variability related to differences across universities. Students with impairments or insufficient Greek-language comprehension were excluded to ensure that all instruments could be completed reliably. Data collection took place over 10 sessions after regular course lectures, during which questionnaires were distributed and completed.

### 2.2 Procedure

At the end of each class, students were provided with a brief explanation of the study and invited to participate. Those who agreed to take part were asked to read and sign a written informed consent form before proceeding. Upon providing written consent, participants received a set of questionnaires as described below. Individuals who chose to withdraw at any stage of the process were excluded from the final data analysis.

### 2.3 Tools

A series of self-report questionnaires was used as detailed below:

(1) A sociodemographic questionnaire, created specifically for this study, to gather essential demographic and medical information through self-reporting. Participants completed questions regarding their gender, age, height, weight, any history of eating disorders, the presence of other psychiatric conditions, medication history and current use, educational background, as well as employment and marital status.

(2) The Eating Disorder Examination Questionnaire (EDE-Q) [45], which is a self-report questionnaire designed as a screening tool to evaluate the severity of symptoms related to eating disorders and various dimensions of eating-related psychopathology over the past 28 days. The EDE-Q includes questions that examine behaviors and attitudes concerning food and body image, using a seven-point Likert scale from 0 to 6, depending on the frequency of behaviors, while some other items assess the frequency of binge eating, vomiting, use of laxatives or diuretics, and excessive exercise in the past four weeks. EDE-Q is scored across four subscales: Restraint, Eating Concern, Shape Concern, and Weight Concern. The mean score of these subscales provides the overall EDE-Q score, with higher scores reflecting more severe eating disorder symptoms [45]. For this study, we used the Greek version of the EDE-Q [46]. The EDE-Q has strong psychometric properties, including high internal consistency and test-retest reliability, and demonstrates good concurrent and criterion validity [47]. Moreover, the Greek version of the EDE-Q also has adequate reliability [48] and validity [46]. The Greek version of the EDE-Q has demonstrated strong internal consistency, with a Cronbach’s alpha of 0.92 for the global scale, and reliability coefficients of 0.78 for the restraint subscale, 0.74 for eating concern, and 0.91 for the combined shape and weight concern subscales [46].

(3) The Binge Eating Scale [49], which is a self-report measure used to identify binge eating behaviors, a core diagnostic criterion for BED. It is a screening tool evaluating both the severity of binge episodes and the effectiveness of related treatments. Comprising 16 questions, the BES examines eight emotional and cognitive aspects of binge eating, such as guilt or concerns about self-control over food intake, while the other eight focus on behavioral indicators, like rapid eating or eating in secrecy. Each item is rated on a Likert scale ranging from 0 to 3 (0 = no binge problem, 3 = severe binge problem) [49]. The total score, calculated by summing all responses, ranges from 0 to 46, with scores of ≤17 indicating no binge eating, 18-26 indicating moderate binge eating, and ≥27 indicating severe binge eating [50]. The BES has strong psychometric characteristics, including high internal consistency, robust construct reliability, convergent validity, and test-retest reliability [51]. The Greek version of the BES was acquired for our study and is currently undergoing standardization by the Eating Disorders Clinic at the 1^st^ Department of Psychiatry at Aiginiteio Hospital (Dr Frangiskos Gonidakis, personal communication). The published validation study confirmed its factorial structure and psychometric properties in a clinical BED population, reporting acceptable internal consistency (Cronbach’s alpha=0.78) and good convergent validity with the EDE-Q and BMI [4].

(4) The Difficulties in Emotion Regulation Scale (DERS) [52], a self-report measure designed to assess difficulties in ER, emphasizing four key dimensions: (a) awareness and understanding of emotions, (b) acceptance of emotions, (c) the capacity to engage in goal-directed behavior and manage impulses during negative emotional experiences, and (d) access to effective, contextually appropriate ER strategies. It consists of 36 items, some of which are reverse-scored, with responses rated from 1 to 5 based on how often each statement applies to the participant. The total score is the sum of all responses, with higher scores reflecting more significant difficulties in ER. The DERS includes six subscales: 1) non-acceptance of emotional responses, 2) challenges in goal-directed behavior, 3) impulse control issues, 4) lack of emotional awareness, 5) restricted access to ER strategies, and 6) lack of emotional clarity [52]. This study used the Greek version of the DERS, which comprises five subscales: Self-Control, Recovery, Clarity, Non-Acceptance, and Awareness Effort [53]. The DERS has strong internal consistency, reliable test-retest results, and sufficient predictive and structural validity [52]. The Greek version also demonstrates satisfactory psychometric properties, with a Cronbach’s alpha of 0.92 [53].

(5) The Morningness-Eveningness Questionnaire (MEQ) [30], which is a self-report questionnaire consisting of 19 questions and is designed for the assessment of the natural sleep-wake cycle and the detection of chronotypes [54]. Each question provides four to six response options. The overall MEQ score is obtained by summing all responses, with scores ranging from 16 to 86; higher scores indicate a morning-oriented type, and lower scores an evening-oriented type. Participants are categorized based on their scores: 70-86 as "Definitely Morning Type," 59-69 as "Moderately Morning Type," 42-58 as "Neither Type," 31-41 as "Moderately Evening Type," and 16-30 as "Definitely Evening Type" [30]. The MEQ has demonstrated strong psychometric properties, including high internal consistency and stability over time [55]. Its validity is supported by comparisons with both objective measures, such as cortisol levels and body temperature, and subjective indicators, such as sleep-wake patterns [56, 57]. The original MEQ has not been translated or previously adapted for use in the Greek population. Thus, for the purposes of this study, standard translation and cultural adaptation procedures, including forward and backward translation by bilingual experts (EK, EMT, AK, KNF), were employed to ensure linguistic and conceptual equivalence. The translated version was tested for comprehensibility and cultural appropriateness, and no modifications were required. In the present sample, the Greek version of MEQ (Supplementary Material) demonstrated good internal consistency with a Cronbach’s alpha of 0.815.

### 2.4 Statistical Analysis

An a priori power analysis was conducted using G*Power version 3.1.9.7 [58] to estimate sample size. Based on previous studies examining similar associations among the study variables [40, 59, 60], the effect size was determined to be 0.20. With a significance level of α = 0.05 and a statistical power of 0.80, the analysis indicated that the minimum sample size needed with this effect size is N = 193. The actual sample size obtained for the current study (N = 269) exceeded this requirement, indicating that the study is adequately powered to test the research hypotheses.

Statistical analysis of the collected data was conducted using the SPSS (Statistical Package for the Social Sciences) Statistics version 29.0. Correlations were evaluated using the non-parametric Spearman’s rho, as the data were not normally distributed. We also used PROCESS SPSS macro [61] to examine possible mediation effects.

## 3 RESULTS

 Descriptive statistics and participant scores on the EDE-Q, BES, DERS, and MEQ are summarized in Tables **1** and **2**, respectively. The mean age (± Standard Deviation, SD) of 269 participants was 21.07 (± 4.36) years, with an age range spanning from 18 to 49 years, while the mean BMI (±SD) was 21.83 (±3.25) kg/m^2^, with a range spanning from 15.6 to 35.7 kg/m^2^ (Table **2**).

Using a cutoff of 2.8 on the EDE-Q scores [62], 23.8% (n=64) of the participants reported symptoms of eating psychopathology, while 76.2% (n=205) did not, indicating that even in a non-clinical university sample, a meaningful subset exceeded the established threshold. According to the BES scores, 78.4% of participants (n=211) did not report binge eating behaviors, 16.7% (n=45) reported moderate binge eating behaviors, and 4.8% (n=13) reported severe binge eating behaviors [50]. According to the DERS scores, 41.3% (n=111) of participants scored below 90, indicating less severe ER difficulties, while 58.7% (n=158) scored above 90, indicating more severe ER difficulties, reflecting that a substantial share of this non-clinical sample reported difficulties above the established threshold [52, 53]. Moreover, based on their MEQ scores, 4.8% (n=13) of participants were classified as morning chronotypes (score ≥59), 54.6% (n=147) as intermediate chronotypes (score 42-58), and 40.5% (n=109) as evening chronotypes (score ≤41) [30] (Table **3**).

 Spearman's correlations were tested between BMI and (i) eating psychopathology, as assessed by EDE-Q, (ii) binge eating behaviors, as measured by BES, (iii) difficulties in ER, as evaluated by DERS, and (iv) chronotypes, as assessed by MEQ (Table **4**). We found a statistically significant positive correlation between BMI and eating psychopathology (r_s_=0.404, *p<*0.001) and binge eating behaviors (r_s_=0.387, *p<*0.001). Eating psychopathology had a statistically significant positive correlation with binge eating behaviors (r_s_=0.723, *p<*0.001) and ER difficulties (r_s_=0.434, *p<*0.001). Furthermore, binge eating had a statistically significant positive correlation with difficulties in ER (r_s_=0.429, *p<*0.001). Lastly, regarding ER difficulties, Spearman's correlation showed a statistically significant association with the evening chronotype (r_s_ = -0.150, *p* = 0.014).

Since binge eating behaviors did not correlate directly with chronotypes, but both showed correlations with ER difficulties, we performed a mediation analysis to further explore these relationships. A mediation analysis, using 5000 bootstrap samples and unstandardized coefficients, and involving ER difficulties as a mediator, revealed that ER difficulties mediated the association between chronotype and binge eating. The path coefficient from chronotype to ER difficulties was statistically significant (a = -0.34, 0.02), indicating that morningness was associated with fewer difficulties in ER. The path coefficient from ER difficulties to binge eating behaviors was also statistically significant (b=0.17, *p<*0.001), indicating that difficulties in ER had a direct impact on binge eating. Even though the direct effect of chronotype on binge eating became non-significant when controlling for the mediator (c'=-0.041, *p*=0.44), the indirect effect of chronotype on binge eating through ER difficulties was significant (Indirect Effect (axb) =-0.06, 95% confidence intervals: -0.11– -0.01). This pattern of results confirms that difficulties in ER fully mediated the relationship between chronotype and binge eating (Table **5**; Fig. **1**).

Given that age and BMI are associated with chronotype [32] and eating behaviors [12], respectively, the mediation analysis was re-estimated while controlling for these covariates. Both the path coefficient from chronotype to ER difficulties (a=-0.36, *p*=0.017) and the path coefficient from ER difficulties to binge eating behaviors (b=0.15, *p<*0.001) remained statistically significant. The direct effect of chronotype on binge eating became non-significant when ER difficulties were included (c′=-0.08, *p*=0.10). However, the indirect effect of chronotype on binge eating through ER difficulties remained significant (Indirect Effect (axb)=-0.056, 95% confidence intervals: -0.10 – -0.01). This pattern reflects a full mediation effect that remained evident even after adjusting for age and BMI (Table **6**; Fig. **2**).

## 4 DISCUSSION

The current study was designed to explore possible relationships between binge eating behaviors, ER difficulties, and chronotypes. We focused on a cohort of female university students, as eating disorders are more prevalent among young women compared to other age groups of the general population [5].

Our study confirmed the association of binge eating behaviors with difficulties in the ER. Women with more severe eating psychopathology, as evaluated by EDE-Q, and more severe binge eating behaviors, as depicted by BES, also experience more ER difficulties. Thus, in line with previous findings, eating psychopathology and binge eating behaviors appear to be closely linked to emotion regulation (ER), with women experiencing greater ER difficulties more likely to report higher levels of binge eating behaviors [5, 60]. We also found an association between evening chronotype and difficulties in ER. This finding aligns with the existing literature supporting the notion that evening chronotypes experience more difficulties in ER [59, 63] compared to other chronotypes. Furthermore, both EDE-Q and BES scores were positively correlated with BMI, suggesting that eating psychopathology and binge eating behaviors are linked to obesity [9].

Despite expectations, the current data do not show a direct association between eating psychopathology, binge eating behaviors, and chronotypes. However, mediation analysis revealed an indirect effect of chronotype on binge eating behaviors *via* difficulties in ER, indicating that eveningness is indirectly associated with binge eating behaviors through its relationship with ER difficulties. Importantly, this indirect pathway persisted even after controlling for age and BMI, which are commonly linked to chronotype [32] and eating behaviors [12], suggesting that the mediating role of ER difficulties cannot be explained solely by demographic or weight-related influences, but may represent a distinct mechanism connecting chronotype to eating patterns. Similarly, Martins and colleagues (2024) [64] found that evening chronotype was associated with greater emotional eating and elevated risk of BED in university students, reinforcing the role of eveningness in shaping disordered eating patterns in young populations. In addition, van den Berg and colleagues (2018) [65] recommended that individuals who are evening-oriented face greater ER difficulties, possibly affecting their eating behaviors as well. In our study, the small mediation effect implies that, within the complex network of factors influencing binge eating, chronotype is likely to play a lesser role. Biological, psychological, and environmental influences collectively shape eating behaviors [66]. While evening chronotype was indirectly associated with binge eating through its link with ER difficulties, this alone is insufficient to explain binge eating patterns comprehensively.

As expected, EDE-Q scores were significantly correlated with BES scores, confirming the association between eating psychopathology and binge eating behaviors [1]. The distribution of binge eating behaviors in our sample aligns closely with findings from similar studies, supporting the BES as a valid tool across non-clinical groups. Similar to our study, Duarte *et al*. (2015) [51] and Escrivá-Martínez *et al*. (2019) [67] used samples predominantly of young, female university students and observed similar patterns in binge-eating severity. These shared characteristics across studies highlight the stability of BES-measured binge eating behaviors in comparable populations, suggesting that the scale can effectively capture binge eating tendencies in young, non-clinical groups, where early detection may facilitate timely intervention.

Our finding of nearly 60% of participants scoring above 90 on the DERS, indicating severe ER difficulties, appears higher than the rates observed in other general population studies [52, 53]. Given that our sample consisted mainly of psychology students, it is plausible that increased awareness of psychological issues in this group [68] could contribute to heightened self-reporting of ER difficulties.

Furthermore, our sample’s low prevalence of morning chronotypes is consistent with the mean age of participants (21.07(± 4.36) years), aligning with established age-related chronotype trends. Research reports that eveningness peaks in late adolescence and early adulthood, around age 20, and gradually shifts toward morningness with age [69]. This well-known pattern supports our finding of most participants falling into intermediate or evening chronotypes, underscoring the influence of age on chronotype distribution. Age-related chronotype tendencies could therefore be a relevant factor in examining the associations among chronotype, eating behaviors, and emotional regulation [70].

 Our finding regarding the indirect association of binge eating behaviors with chronotypes in our sample, aligns with the study by Esin and Ayyildiz (2024) [71], who also, found no relationship between chronotypes, as measured by the MEQ, and eating disorder risk, assessed using the SCOFF questionnaire, in a sample of university students with an average age of 20 years. However, several studies suggest that an evening chronotype is associated with disordered eating patterns [72] and BED [40, 41, 73, 74]. Differences in our findings compared to these studies could stem from key factors in study design and sample characteristics.

For example, Harb and colleagues (2012) [40] and Mason and colleagues (2019) [73] studied older populations, where stable routines likely enhance the effect of chronotype on eating patterns. Mason and colleagues’ (2019) [73] broader psychopathological sample, and Romo-Nava and colleagues’ (2020) [41] clinical focus on bipolar disorder, both amplify circadian and eating disturbances. López-Gil and colleagues (2024) [74] studied adolescents and identified a direct association between evening chronotype and disordered eating, likely influenced by heightened social jetlag, common in adolescent populations, and by the SCOFF questionnaire, which captures a broad range of disordered eating behaviors. In contrast, our young, healthy sample of university students may lack these developmental or clinical factors, leading to a subtler, indirect relationship mediated by ER. Previous studies also used targeted eating behavior assessments, such as the Night Eating Questionnaire [40] or adolescent-specific tools, such as the Morningness/ Eveningness Scale in Children [74], which may capture stronger connections. In sum, clinical, adolescent, or older populations may show a direct effect of eveningness on disordered eating, whereas in young, healthy groups, the link may be mediated by other factors, such as ER.

Additionally, in accordance with some studies [40, 71], we found no association between chronotypes and BMI. However, other studies have shown varying results. Merikanto and colleagues (2014) [75] suggested that individuals with an evening chronotype have a lower BMI, whereas other studies found that individuals with a morning chronotype usually have a normal BMI [76] and that those with an evening chronotype are more likely to be overweight or obese [39, 76].

Moreover, Galasso *et al*. (2019) [77] observed alterations in the circadian system of BED patients, indicating a potential association between binge eating behaviors and circadian rhythm disturbances. Interestingly, the hypothesis that circadian rhythm regulation may improve disordered eating patterns emerged in a recent study [71].

Chronotherapy, which focuses on stabilizing the circadian system, has shown potential in supporting the remission of eating psychopathology [78]. By systematically adjusting light exposure, sleep-wake timing, and daily routines, chronotherapy aims to realign an individual’s biological clock with a regular 24-hour rhythm, enhancing circadian alignment and improving related symptoms [79]. However, these findings remain somewhat inconsistent, underscoring the need for further research to clarify the effectiveness of chronotherapeutic interventions for eating disorders and to identify the factors that might influence individual responses [72].

### 4.1 Limitations

However, our results should be interpreted with caution. Considering the number of variables, our sample was small. Another significant limitation is the cross-sectional design, which precludes establishing causal relationships between variables. Moreover, the use of a convenience sample of university students restricts our ability to generalize the findings to broader populations. A further limitation is that only female students were recruited. Although this decision was based on the higher prevalence of binge eating behaviors in women, it restricts the generalizability of our findings to male populations. Most participants were psychology students, although the exact number was not recorded. This introduces a potential bias, as psychology students may be more aware of their psychological states than other populations [68]. Our sample may therefore reflect specific reporting tendencies related to the participants’ field of study, which should be considered when generalizing the findings to other populations.

Another notable limitation of our study is the absence of measures of sleep duration and quality. Previous research has consistently indicated that the disadvantages faced by evening-type individuals often arise from a misalignment with societal schedules, leading to both chronic social jetlag and persistent sleep deprivation [80, 81]. In our student cohort, however, morning demands may be less stringent than in typical work environments, potentially mitigating the impact of chronotype misalignment. Assessing sleep patterns would have provided valuable insights into this aspect, but unfortunately, these data were not available in our study.

### 4.2 Future Research

Suggestions for future research include increasing the sample size and enhancing the diversity of participants by age and field of study. Moreover, binge eating behaviors should be studied in men, since BED has a high prevalence in males as well [1, 2, 4]. Furthermore, our study could be applied to clinical populations as well, while future research should also focus on how ER and chronotypes associate with other eating disorders, such as anorexia nervosa and bulimia nervosa. Future studies could also focus on eating psychopathology in LGBTQ+ individuals, who have been shown to have an increased frequency of eating disorders [82-84]. Future research should also adopt longitudinal designs to clarify the temporal and potentially causal relationships between chronotype, ER, and binge eating behaviors. Interventional studies should explore whether approaches targeting ER or circadian alignment (*e.g*., light therapy, chronotherapy, or cognitive-behavioral strategies) may help in reducing binge eating behaviors. Finally, future studies could integrate biological markers, such as cortisol rhythms, inflammatory markers, or genetic polymorphisms, to elucidate underlying neurobiological mechanisms.

## CONCLUSION

This study did not show a direct association between chronotype and binge eating behaviors. However, we observed a strong direct association between ER and binge eating and found that ER difficulties statistically mediated the association between chronotype and binge eating. This study emphasizes the importance of ER and its potential connection with chronotype. These findings suggest that ER may represent a potential intervention target, which future studies could explore in combination with chronotherapy to address circadian misalignment commonly seen in individuals with late chronotypes.

## Figures and Tables

**Fig. (1):**
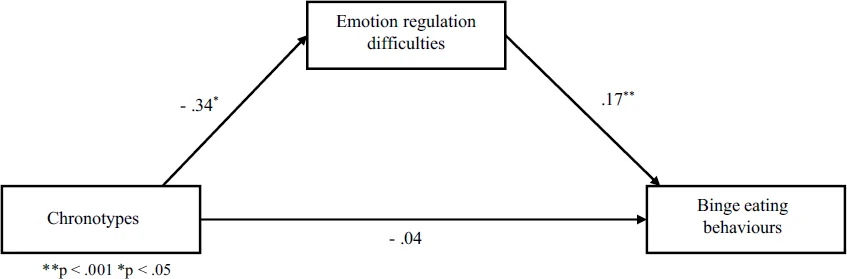
Mediation model showing the role of emotion regulation difficulties as a mediator between chronotype and binge eating behaviours.

**Fig. (2):**
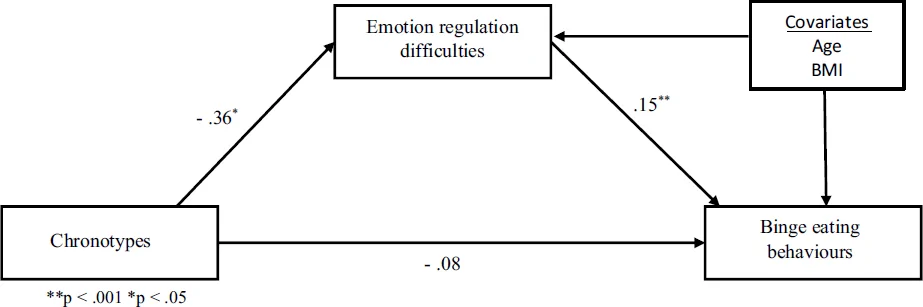
Mediation model showing the role of emotion regulation difficulties as a mediator between chronotype and binge eating behaviours after controlling for age and BMI.

**Table 1:** Sociodemographic characteristics of participants.

**-**	**n**	**%**
**Highest Educational Level**		
High school	247	91.8
Bachelor	12	4.5
Postgraduate degree	9	3.3
Other	1	.4
**Employment**		
Only students	236	87.7
Part time job	23	8.6
Full time job	9	3.3
Retired	1	.4
**Marital Status**		
Single	154	57.2
In relationship	108	40.1
Married	6	2.2
Widowed	1	.3
**BMI**		
Underweight, <19	39	14.6
Normal, 19-24	190	70.9
Overweight/Obese, ≥25	39	14.6
BED diagnosis	5	1.9
Other psychiatric diagnosis	30	11.2
Depression	14	5.2
Anxiety Disorder	4	1.5
Generalized Anxiety Disorder	4	1.5
Obsessive Compulsive Disorder	3	1.1
Anorexia Nervosa	2	.7
Attention Deficit Disorder	1	.4
Agoraphobia	1	.4
Autism	1	.4
Bipolar Disorder	1	.4
Borderline Personality Disorder	1	.4
Mixed Anxiety Depressive Disorder	1	.4
Nosophobia	1	.4
Panic Disorder	1	.4
Social Anxiety Disorder	1	.4
Depression with psychotic symptoms	1	.4
Psychotic Disorder	1	.4
Medication	27	10
Contraceptives	10	3.7
Treatment for thyroid disorders	7	2.6
Antidepressants	6	2.2
Vitamins	3	1.2
Acne treatment	2	.7
Antipsychotics	2	.7
Αntihypertensive medication	2	.7
Anxiolytics	1	.4
Benzodiazepines	1	.4

**Note**: N=269 except for BMI, where N=268 Abbreviation: BMI, Body Mass Index.

**Table 2:** Descriptive statistics of age and EDE-Q, BES, DERS, MEQ scores.

-	**Mean**	**SD**	**Min.**	**Max.**
Age	21.07	4.36	18	49
BMI	21.83	3.25	15.6	35.7
EDE-Q score	1.69	1.28	0	5
BES score	10.96	8.38	0	42
DERS score	94.97	21.29	44	148
MEQ score	44.02	8.72	21	67

**Note**: N=269 except for BMI, where N=268.

**Abbreviations**: BMI, Body Mass Index; EDE-Q, Eating Disorder Examination Questionnaire; BES, Binge Eating Scale; DERS, Difficulties in Emotion Regulation Scale; MEQ, Morningness-Eveningness Questionnaire.

**Table 3:** Classification of the participants according to their EDE-Q, BES, DERS and MEQ scores.

**-**	**n**	**%**
**Eating Psychopathology (EDE-Q)**		
Yes, ≥ 2.8	64	23.8
No, < 2.8	205	76.2
**Binge Eating (BES)**		
No binge eating, ≤ 17	211	78.4
Moderate binge eating, 18-26	45	16.7
Severe binge eating, ≥ 27	13	4.8
**Difficulties in Emotion Regulation (DERS)**		
Less severe emotion regulation difficulties, ≤ 89	111	41.3
More severe emotion regulation difficulties, ≥ 90	158	58.7
**Chronotypes (MEQ)**		
Morning chronotype, ≥ 59	13	4.8
Intermediate chronotype, 42-58	147	54.6
Evening chronotype, ≤41	109	40.5

**Note**: N=269.

**Abbreviations**: EDE-Q, Eating Disorder Examination Questionnaire; BES, Binge Eating Scale; DERS, Difficulties in Emotion Regulation Scale; MEQ, Morningness-Eveningness Questionnaire.

**Table 4:** Spearman correlations between ΒΜΙ, total scores of EDE-Q, BES, DERS, MEQ and scores of EDE-Q and DERS subscales.

-	**1**	**2**	**3**	**4**	**5**	**6**	**7**	**8**	**9**	**10**	**11**	**12**	**13**	**14**
1. BMI	-	-	-	-	-	-	-	-	-	-	-	-	-	-
2. EDE-Q total score	.404**	-	-	-	-	-	-	-	-	-	-	-	-	-
3. BES total score	.387**	.723**	-	-	-	-	-	-	-	-	-	-	-	-
4. DERS total score	.108	.434**	.429**	-	-	-	-	-	-	-	-	-	-	-
5. MEQ total score	.109	-.085	-.103	-.150*	-	-	-	-	-	-	-	-	-	-
6. EDE-Q Restraint	.355**	.789**	.546**	.269**	.004	-	-	-	-	-	-	-	-	-
7. EDE-Q Eating Concern	.237**	.822**	.700**	.415**	-.156*	.581**	-	-	-	-	-	-	-	-
8. EDE-Q Shape Concern	.383**	.943**	.684**	.429**	-.118	.651**	.713**	-	-	-	-	-	-	-
9. EDE-Q Weight Concern	.425**	.929**	.673**	.428**	-.097	.617**	.734**	.884**	-	-	-	-	-	-
10. DERS Self Control	.041	.367**	.390**	.883**	-.185**	.233**	.348**	.367**	.361**	-	-	-	-	-
11. DERS Recovery	-.017	.279**	.319**	.771**	-.168**	.139*	.285**	.280**	.277**	.729**	-	-	-	-
12. DERS Clarity	.143*	.253**	.270**	.634**	-.034	.154*	.273**	.235**	.257**	.344**	.376**	-	-	-
13. DERS Non-Acceptance	.056	.394**	.337**	.752**	-.106	.263**	.376**	.384**	.397**	.600**	.429**	.372**	-	-
14. DERS Awareness Effort	.101	.161**	.129*	.356**	.050	.076	.180**	.153*	.154*	.089	.206**	.474**	.132*	-

**Note**: **p < .01. *p < .05, N=269 except for BMI, where N=268.

**Abbreviations**: BMI, Body Mass Index; EDE-Q, Eating Disorder Examination Questionnaire; BES, Binge Eating Scale; DERS, Difficulties in Emotion Regulation Scale; MEQ, Morningness-Eveningness Questionnaire.

**Table 5:** Mediation of emotion regulation difficulties in the association between chronotypes and binge eating behaviours.

**Path**	**Predictor → Outcome**	**B**	**SE**	**t**	***p***	**95% CI (LL, UL)**
a	Chronotype → ER difficulties	-0.35	0.15	-2.34	.020	-0.64, -0.05
b	ER difficulties → BEBs	0.17	0.02	7.77	<.001	0.13, 0.21
c′	Chronotype → BEBs (direct effect)	-0.04	0.05	-0.78	.437	-0.15, 0.06
Indirect Effect (a×b)	Chronotype → ER → BEBs	-0.06	0.03	-	-	-0.11, -0.01

**Note**: N = 269, 5,000 bootstrap samples.

**Abbreviations**: ER, Emotion Regulation; BEBs, Binge Eating Behaviours.

**Table 6:** Mediation of emotion regulation difficulties in the association between chronotype and binge eating behaviours, adjusted for age and BMI.

**Path**	**Predictor → Outcome**	**B**	**SE**	**t**	***p***	**95% CI (LL, UL)**
a	Chronotype → ER difficulties	-0.36	0.15	-2.41	.017	-0.66, -0.07
a	BMI → ER difficulties	0.70	0.40	1.74	.083	-0.09, 1.49
a	Age → ER difficulties	-0.04	0.30	-0.14	.889	-0.64, 0.56
b	ER difficulties → BEBs	0.15	0.02	7.57	<.001	0.11, 0.19
b	BMI → BEBs	0.98	0.13	7.41	<.001	0.72, 1.24
b	Age → BEBs	-0.05	0.10	-0.46	.648	-0.24, 0.15
c′	Chronotype → BEBs (direct effect)	-0.08	0.05	-1.65	.100	-0.18, 0.02
Indirect Effect (a×b)	Chronotype → ER → BEBs	-0.056	0.024	-	-	-0.10, -0.01

**Note**: N = 268, 5,000 bootstrap samples.

**Abbreviations**: ER, Emotion Regulation; BEBs, Binge Eating Behaviours; BMI, Body Mass Index.

## Data Availability

All data generated or analyzed during this study are included in this published article.

## LIST OF ABBREVIATIONS

BED: Binge Eating Disorder
BMI: Body Mass Index
ER: Emotion Regulation

## STANDARDS OF REPORTING

STROBE guidelines were followed.
