# Supplementary material for: Associations Among Binge Eating Behaviors, Emotion Regulation, and Chronotype in Female Greek University Students
Source: Curr Neuropharmacol. 2026 Jan 13;24(11):1765–76. doi: 10.2174/011570159X431924251127110942 (PMC13647652; doi:10.2174/011570159X431924251127110942)
Supplement: Supplementary file 1 [file CN-24-11-1765_SD1.pdf]

Supplementary Material

Associations Among Binge Eating Behaviors, Emotion Regulation, and Chronotype in Female Greek University Students

Efi Karakosta[1,*], Evangelia-Maria Tsapakis[1], Anastasia Konsta[2], Anat Lan[3], Haim Einat[1,3] and Konstantinos N. Fountoulakis[1]

[1] 3rd Department of Psychiatry, School of Medicine, Faculty of Health Sciences, Aristotle University of Thessaloniki, Thessaloniki, Greece; [2] 1st Department of Psychiatry, Papageorgiou General Hospital of Thessaloniki, Aristotle University of Thessaloniki, Thessaloniki, Greece; [3] School of Behavioral Sciences, Tel Aviv-Yaffo Academic College, Tel Aviv, Israel

MORNINGNESS-EVENINGNESS QUESTIONNAIRE (MEQ)

Instructions:
- Please read each question very carefully before answering.
- Please answer each question as honestly as possible.
- Answer ALL questions.
- Each question should be answered independently of others. Do NOT go back and check your answers.

1. What time would you get up if you were entirely free to plan your day?

5:00 – 6:30 AM	5
6:30 – 7:45 AM	4
7:45 – 9:45 AM	3
9:45 – 11:00 AM	2
11:00 AM – 12 NOON	1
12 NOON – 5:00 AM	0

2. What time would you go to bed if you were entirely free to plan your evening?

8:00 – 9:00 PM	5
9:00 – 10:15 PM	4
10:15 PM – 12:30 AM	3
12:30 – 1:45 AM	2
1:45 – 3:00 AM	1
3:00 AM – 8:00 PM	0

3. If there is a specific time at which you have to get up in the morning, to what extent do you depend on being woken up by an alarm clock?

Not at all dependent	4
Slightly dependent	3
Fairly dependent	2
Very dependent	1

4. How easy do you find it to get up in the morning (when you are not woken up unexpectedly)?

Not at all easy	1
Not very easy	2
Fairly easy	3
Very easy	4

1875-6190/26 $65.00+.00 © 2026 Bentham Science Publishers

5. How alert do you feel during the first half hour after you wake up in the morning?

Not at all alert	1
Slightly alert	2
Fairly alert	3
Very alert	4

6. How hungry do you feel during the first half-hour after you wake up in the morning?

Not at all hungry	1
Slightly hungry	2
Fairly hungry	3
Very hungry	4

7. During the first half-hour after you wake up in the morning, how tired do you feel?

Very tired	1
Fairly tired	2
Fairly refreshed	3
Very refreshed	4

8. If you have no commitments the next day, what time would you go to bed compared to your usual bedtime?

Seldom or never later	4
Less than one hour later	3
1-2 hours later	2
More than two hours later	1

9. You have decided to engage in some physical exercise. A friend suggests that you do this for one hour twice a week and the best time for him is between 7:00 – 8:00 am. Bearing in mind nothing but your own internal "clock", how do you think you would perform?

Would be in good form	4
Would be in reasonable form	3
Would find it difficult	2
Would find it very difficult	1

10. At what time of day do you feel you become tired as a result of need for sleep?

8:00 – 9:00 PM	5
9:00 – 10:15 PM	4
10:15 PM – 12:45 AM	3
12:45 – 2:00 AM	2
2:00 – 3:00 AM	1

11. You want to be at your peak performance for a test that you know is going to be mentally exhausting and will last for two hours. You are entirely free to plan your day. Considering only your own internal "clock", which ONE of the four testing times would you choose?

8:00 AM – 10:00 AM	4
11:00 AM – 1:00 PM	3
3:00 PM – 5:00 PM	2
7:00 PM – 9:00 PM	1

12. If you got into bed at 11:00 PM, how tired would you be?

Not at all tired	1
A little tired	2
Fairly tired	3
Very tired	4

13. For some reason you have gone to bed several hours later than usual, but there is no need to get up at any particular time the next morning. Which ONE of the following are you most likely to do?

Will wake up at usual time, but will NOT fall back asleep	4
Will wake up at usual time and will doze thereafter	3
Will wake up at usual time but will fall asleep again	2
Will NOT wake up until later than usual	1

14. One night you have to remain awake between 4:00 – 6:00 AM in order to carry out a night watch. You have no commitments the next day. Which ONE of the alternatives will suite you best?

Would NOT go to bed until watch was over	1
Would take a nap before and sleep after	2
Would take a good sleep before and nap after	3
Would sleep only before watch	4

15. You have to do two hours of hard physical work. You are entirely free to plan your day and considering only your own internal "clock" which ONE of the following time would you choose?

8:00 AM – 10:00 AM	4
11:00 AM – 1:00 PM	3
3:00 PM – 5:00 PM	2
7:00 PM – 9:00 PM	1

16. You have decided to engage in hard physical exercise. A friend suggests that you do this for one hour twice a week and the best time for him is between 10:00 – 11:00 PM. Bearing in mind nothing else but your own internal "clock" how well do you think you would perform?

Would be in good form	1
Would be in reasonable form	2
Would find it difficult	3
Would find it very difficult	4

17. Suppose that you can choose your own work hours. Assume that you worked a FIVE hour day (including breaks) and that your job was interesting and paid by results). Which FIVE CONSECUTIVE HOURS would you select?

5 hours starting between 4:00 AM and 8:00 AM	5
5 hours starting between 8:00 AM and 9:00 AM	4
5 hours starting between 9:00 AM and 2:00 PM	3
5 hours starting between 2:00 PM and 5:00 PM	2
5 hours starting between 5:00 PM and 4:00 AM	1

18. At what time of the day do you think that you reach your "feeling best" peak?

5:00 – 8:00 AM	5
8:00 – 10:00 AM	4
10:00 AM – 5:00 PM	3
5:00 – 10:00 PM	2
10:00 PM – 5:00 AM	1

19. One hears about "morning" and "evening" types of people. Which ONE of these types do you consider yourself to be?

Definitely a "morning" type	6
Rather more a "morning" than an "evening" type	4
Rather more an "evening" than a "morning" type	2
Definitely an "evening" type	0
